# Tumor Necrosis Factor Receptor 1 Regulates Colonic Mesenchymal Cell Diversity and the Epithelial Stem Cell Niche

**DOI:** 10.1016/j.jcmgh.2026.101856

**Published:** 2026-08-11

**Authors:** Safina Gadeock, Nandini Girish, Cambrian Y. Liu, Ying Huang, Barun Das, Tracy C. Grikscheit, D. Brent Polk

**Affiliations:** 1 Division of Pediatric Gastroenterology, Hepatology, and Nutrition, and The Saban Research Institute, Children’s Hospital Los Angeles, Los Angeles, California; 2 Department of Pediatrics, University of California San Diego School of Medicine, La Jolla, California; 3 Department of Medicine, The University of Chicago, Chicago, Illinois; 4 Department of Surgery, Keck School of Medicine, University of Southern California, Los Angeles, California; 5 Department of Biochemistry and Molecular Medicine, Keck School of Medicine, University of Southern California, Los Angeles, California; 6 Division of Gastroenterology, Hepatology, and Nutrition, Department of Pediatrics, Rady Children’s Hospital, San Diego, San Diego, California

**Keywords:** IBD Organoids, Mesenchyme, Stem Cell Niche, TNF

## Abstract

**Background & Aims:**

Tumor necrosis factor receptor 1 regulates intestinal epithelial survival and repair, yet its role in the colonic mesenchyme remains unclear. We investigated how mesenchymal tumor necrosis factor receptor 1 signaling controls stromal cell identity, stem cell niche function, and epithelial homeostasis.

**Methods:**

Colonic mesenchymal populations from germline tumor necrosis factor receptor 1–deficient (TNFR1^−/−^) and mesenchymal-specific Pdgfra-Cre;TNFR1^fl/fl^ mice were analyzed using immunostaining, Western blotting, bulk and single-cell RNA sequencing, and organoid–mesenchymal coculture systems. Functional studies included wound healing assays, recombinant R-spondin 3 rescue, and integrin A6 blockade.

**Results:**

Tumor necrosis factor receptor 1 deletion altered mesenchymal composition, reducing platelet derived growth factor receptor α expression while increasing α smooth muscle actin expression in vivo and in vitro. Single-cell RNA sequencing demonstrated loss of mesenchymal heterogeneity following tumor necrosis factor receptor 1 deletion, including depletion of a distinct tumor necrosis factor– and interferon-responsive mesenchymal subpopulation enriched for Mmp9, Cd200, Traf1, and Tnfrsf9. Transcriptomic profiling revealed downregulation of stem cell niche factors including Rspo3, Wnt2b, and Fgf2, alongside increased extracellular matrix and proliferative signaling pathways, particularly integrin A6, phosphoinositide 3-kinase, mitogen-activated protein kinase, and Ras-associated protein 1 signaling. Mesenchymal tumor necrosis factor receptor 1 deletion impaired epithelial proliferation and reduced Lgr5+ and Ephb3+ intestinal stem cell expression in vivo. In organoid coculture systems, tumor necrosis factor receptor 1–deficient mesenchymal cells suppressed epithelial stem cell marker expression and altered colonoid morphology, effects which were rescued by recombinant R-spondin 3. Integrin A6 blockade restored platelet derived growth factor receptor α and R-spondin 3 expression and normalized proliferative signaling and migration in tumor necrosis factor receptor 1–deficient myofibroblasts. Similarly, tumor necrosis factor receptor 1–dependent regulation of integrin A6, platelet derived growth factor receptor α, and R-spondin 3 was observed in human colonic myofibroblasts.

**Conclusions:**

Mesenchymal tumor necrosis factor receptor 1 preserves colonic mesenchymal diversity, sustains R-spondin 3–dependent stem cell niche signaling, and maintains intestinal stem cell homeostasis.

## Graphical abstract

**Figure:**
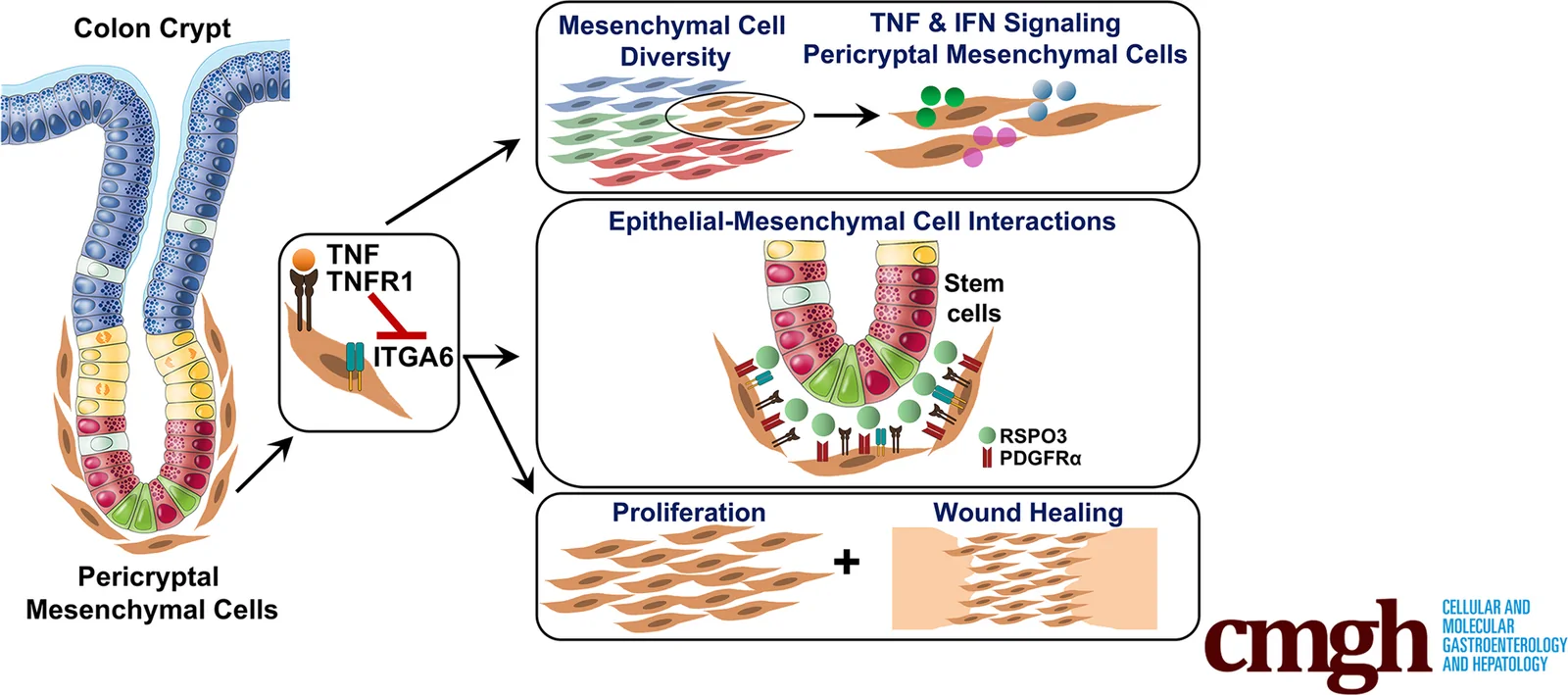

**Summary**

Tumor necrosis factor–tumor necrosis factor receptor 1 signaling by pericryptal platelet-derived growth factor receptor alpha+ mesenchymal cells maintain colonic leucine-rich repeat-containing G protein-coupled receptor 5+ epithelial stem cell numbers, overall colonic mesenchymal cell diversity, and a distinct sub-population of pericryptal mesenchymal cells enriched for tumor necrosis factor and interferon signaling genes.

**What You Need to Know**

**Background**

Tumor necrosis factor (TNF) signaling has dual roles in inflammatory bowel disease (IBD), driving both epithelial damage and wound repair. This study examined how tumor necrosis factor receptor 1 (TNFR1) on mesenchymal cells shapes the epithelial stem cell niche, using TNFR1-deficient mice, single-cell RNA-sequencing, and organoid-mesenchymal cocultures.

**Impact**

This work identifies mesenchymal TNFR1 as a key regulator of the stem cell niche, revealing that TNF signaling supports epithelial regeneration not only through direct effects on the epithelium but also indirectly through the surrounding mesenchyme. By demonstrating that this niche-support function is reversible, the study reframes how TNF-targeted therapies may influence mucosal healing in IBD, highlighting the mesenchymal compartment as a previously underappreciated determinant of treatment response and a potential avenue for improving outcomes in patients who fail to heal on current anti-TNF therapies.

**Future Directions**

Future studies should test whether this axis is disrupted in human IBD (especially anti-TNF nonresponders), explore niche factor–based adjuncts to anti-TNF therapy, and map which fibroblast subpopulations drive this support.

Inflammatory bowel diseases (IBDs) are associated with elevated circulating and mucosal levels of cytokines, including tumor necrosis factor (TNF), a central mediator of disease pathogenesis.^1^ TNF’s pathogenic role is underscored by the initial success of anti-TNF monoclonal antibodies, which reduce symptoms and promote mucosal healing in patients with ulcerative colitis or Crohn’s disease.^1^ However, despite these early clinical benefits, the majority of patients lose response, reflecting limitations in our understanding of how anti-TNF therapies work and why they fail.^1^^,^^2^

TNF exerts its effects through 2 receptors, tumor necrosis factor receptor (TNFR)1 (p55, *Tnfrsf1a*) and TNFR2 (p75, *Tnfrsf1b*). TNFR1 is widely expressed throughout the intestinal mucosa, including in epithelial and stromal compartments, whereas TNFR2 is primarily expressed on immune cell subsets and is upregulated in epithelial cells in human IBD and in mouse models of colitis.^2^, ^3^, ^4^ TNF signaling is highly pleiotropic: it drives proinflammatory pathways that promote autoimmune tissue damage and apoptosis,^5^, ^6^, ^7^ yet paradoxically it can also activate programs supporting cell survival,^8^ epithelial regeneration,^9^^,^^10^ and immunologic tolerance that limit colonic inflammation.^11^ These dual functions, together with evidence that colitis is exacerbated in multiple models of TNF pathway ablation^12^^,^^13^ and the clinical observation that anti-TNF therapy fails in most patients,^4^ illustrate the complexity of TNF biology in IBD. Despite its central role, the tissue- and cell-specific contributions of TNFR1 and TNFR2 to intestinal homeostasis and disease remain poorly understood, limiting our ability to predict or improve therapeutic outcomes. A more precise understanding of how TNF signaling operates in distinct intestinal compartments is therefore critical to decipher both its pathogenic and protective functions and to guide strategies for more effective, targeted interventions.

Mesenchymal stromal cells have been implicated in several processes central to mucosal healing^14^ and IBD-associated inflammation.^15^ The colonic myofibroblast population, which harbors a specialized pericryptal mesenchymal subpopulation, provides critical molecular cues (eg, Wnt ligands, transforming growth factors, insulin growth factors, Notch ligands, epidermal growth factors, and Bmp-type molecules) that maintain the self-renewal of crypt stem cells and guide the proper differentiation of epithelial cells along the crypt vertical axis.^14^^,^^16^ During epithelial wound healing, mesenchymal cells secrete noncanonical Wnt ligands that drive crypt morphogenesis and cell proliferation.^17^^,^^18^ Thus, the function of mesenchymal cells is intimately associated with epithelial regenerative capacity in homeostasis and during states of challenge. Whether cytokines associated with IBD might regulate mesenchymal cell behaviors, and consequently the epithelial stem cell pool, remains to be determined, as it is primarily considered that cytokines act cell-autonomously on epithelium. In fact, recent single-cell profiling has demonstrated that the colonic mesenchyme undergoes significant remodeling in IBD,^15^^,^^19^, ^20^, ^21^ which would suggest that the inflammatory milieu has a profound ability to regulate the specification and function of colonic myofibroblasts.

Here, we hypothesized that TNF signaling through TNFR1 regulates the function and niche-supporting capacity of colonic myofibroblasts. Using a PDGFRαCre-mediated TNFR1 deletion model, which predominantly targets mesenchymal populations, together with single-cell transcriptional profiling and mesenchymal–epithelial coculture assays, we found that TNFR1 signaling contributes to the maintenance of colonic myofibroblast populations and supports epithelial stem cell maintenance in the colon. These findings suggest that stromal TNFR1 signaling is an important regulator of epithelial homeostasis and raise the possibility that similar pathways may be modulated during anti-TNF therapy.

## Results

### Mesenchymal Tumor Necrosis Factor Receptor 1 Deletion Alters Colonic Mesenchymal Cell Marker Expression

TNFR1 promotes intestinal epithelial cell survival,^8^ stem cell proliferation,^10^ colonic barrier integrity, mucosal immune cell function, and repair pathways to restrict IBD-associated inflammation.^11^ Its role in the intestinal mesenchyme, however, remains unknown. To investigate the role of TNFR1 in mesenchymal cell specification and function, we compared mesenchymal cell populations in the colonic submucosa of TNFR1^−/−^ and control (TNFR1^+/−^) mice. The colonic submucosa is composed of a heterogeneous mixture of cell types, including endothelial cells, immune/inflammatory cells, and a diverse population of mesenchymal cells. Mesenchymal subtypes can be classified by the expression of markers such as α-smooth muscle actin (αSMA), vimentin (VIM), platelet-derived growth factor receptor alpha (PDGFRα), GLI family zinc finger 1 (GLI1), and/or forkhead box L1 (FOXL1), which collectively identify pericryptal fibroblasts, myofibroblasts, and other stromal populations. We characterized mesenchymal cell populations using immunostaining and quantitative analysis, supported by Western blotting to confirm marker expression at the protein level. In TNFR1^−/−^ mouse submucosa compared with littermate controls, we found a shift in mesenchymal cell markers—a 40% reduction in VIM along with a 58% increase in αSMA expression (Figure 1*AI*, *AII*, *BI*, and *BII*). Pericryptal mesenchymal cells, previously shown to serve as a stem cell niche,^22^ here identified a 30% reduction in expression of PDGFRα in TNFR1^−/−^ mice (Figure 1*AIII* and *BIII*) compared with littermate controls. However, there was no difference in the expression of GLI1 or FOXL1 (Figure 1*AIV*, *AV*, *BIV*, and *BV*).

**Figure 1:**
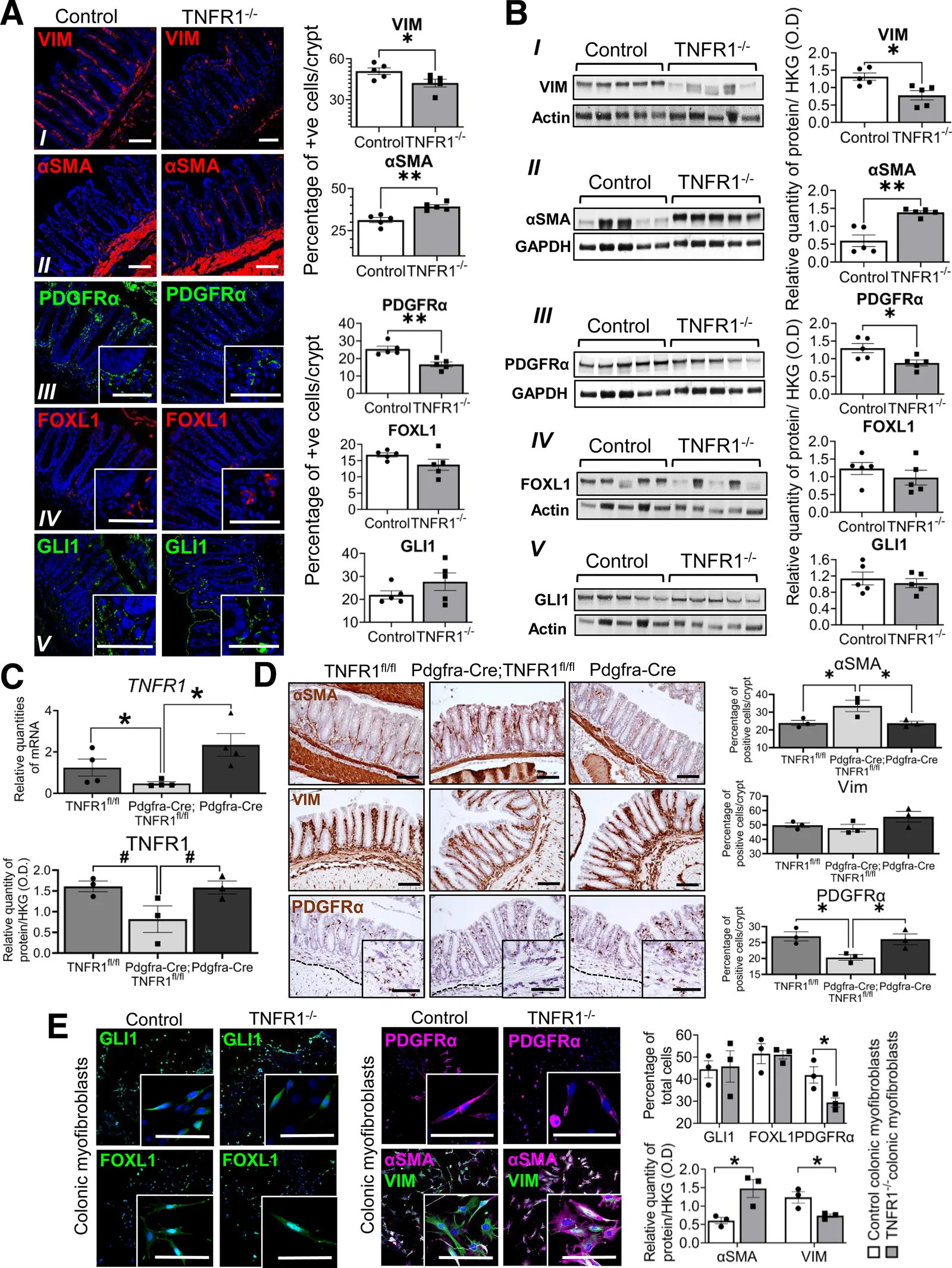
**Mesenchymal TNFR1 deletion alters colonic mesenchymal marker expression.** (*A*) Immunolocalization and percentage of mesenchymal cell (MSC) markers, and (*B*) total proteins levels of (*I*) vimentin, (*II*) αSMA, (*III*) PDGFRα, (*IV*) FOXL1, and (*V*) GLI1 in subepithelial MSCs of the distal colon of 16-week-old control (TNFR1^+/−^) and TNFR1^−/−^ mice. (*C*) Validation of TNFR1 deletion in the primary colonic mesenchyme of Pdgfra-Cre;TNFR1^fl/fl^ mice was performed by measuring total TNFR1 RNA and protein levels relative to TNFR1^fl/fl^ and Pdgfra-Cre control animals. (*D*) Immunolocalization and the percentage of MSC markers, αSMA, VIM, and PDGFRα in the subepithelial and pericryptal niche of distal colon of 6-week-old Pdgfra-Cre;TNFR1^fl/fl^ mice, relative to TNFR1^fl/fl^ and Pdgfra-Cre controls. The *black dotted line* indicates the border of the subepithelial mesenchymal compartment used for analysis. *Subsets* represent magnified localization of the markers in the pericryptal niche. Each *dot* represents an individual biological replicate (number), with 15–20 crypts averaged from the distal colon adjacent to the anal region. (*E*) Immunolocalization and quantification of GLI1, FOXL1, PDGFRα+ cells, and αSMA and VIM expression in control or TNFR1^−/−^ colonic myofibroblasts. Scale bars, 50 μm. All images are representative of n = 3 animals and were taken using a Zeiss LSM 710 Inverted Confocal Microscope (Zeiss). Error bars are presented as the mean ± the standard error of the mean. Data are analyzed by an unpaired Student *t* test or a 1-way analysis of variance, where ∗*P* < .05 and ^#^*P* = .05–.09.

To determine if the specific expression of TNFR1 on the subepithelial mesenchyme alters specification of mesenchymal populations, we generated Pdgfra-Cre;TNFR1^fl/fl^ animals (Figure 1*C*) to ablate TNFR1 in subepithelial PDGFRα+ cells. We show that, although αSMA+ cells increased by 40%, pericryptal PDGFRα+ cells were downregulated by 25% in Pdgfra-Cre;TNFR1^fl/fl^ animals relative to TNFR1^fl/fl^ and Pdgfra-Cre controls (Figure 1*D*).

We next asked whether key mesenchymal subpopulations could be recovered in vitro. Colonic mesenchymal cells from TNFR1^−/−^ and control (TNFR1^+/−^) mice were cultured for 5 days in 3-dimensional conditions. The resulting cultures were immunostained for mesenchymal markers, including VIM, αSMA, PDGFRα, GLI1, and FOXL1 (Figure 1*E*) with cultures demonstrating partially overlapping colonic myofibroblasts populations expressing all markers. Importantly, consistent with observations in TNFR1^−/−^ mice in vivo, the proportion of PDGFRα+ mesenchymal cells within colonic myofibroblasts was reduced by 21% in TNFR1^−/−^-derived mesenchymal cells compared with control (Figure 1*E*). Western blot analysis of mesenchymal cell homogenates confirmed increased αSMA and decreased VIM expression in TNFR1^−/−^ colonic myofibroblasts (Figure 1*E*). These findings indicate that the in vitro culture system recapitulates key features of TNFR1-dependent regulation of colonic myofibroblast composition, supporting its use to study mesenchymal cell identity and function.

### Tumor Necrosis Factor Receptor 1 Is Required to Preserve Colonic Mesenchymal Cell Diversity, Including a Novel Subpopulation Defined by Tumor Necrosis Factor and Interferon Signaling Genes

Because we determined that both germline and PDGFRα-specific deletion of TNFR1 results in significant alterations of mesenchymal cell expression profiles in vivo and in vitro, we hypothesized that TNFR1 regulates colonic myofibroblast populations. To test this prediction, we isolated and expanded myofibroblasts from 3 independent littermate TNFR1^+/−^ (control) and TNFR1^−/−^ mice, followed by single-cell RNA sequencing (scRNA-seq). All cultures were prepared and sequenced concurrently as a single batch, generating >34,000 cells across 6 samples (Figure 2*A*). Clustering identified 6 distinct cell populations (Figure 2*AI*). TNFR1 deficiency substantially reduced mesenchymal heterogeneity, with 4 control clusters collapsing into 2 TNFR1-deficient clusters (Figure 2*AII*). Control-specific clusters (2, 3, 5, and 6) were absent, whereas TNFR1-deficient–specific clusters (1 and 4) emerged consistently across all cultures, indicating that TNFR1 loss reshapes colonic myofibroblast cell type specification and promotes novel mesenchymal populations. We next mapped mesenchymal markers across clusters. Krt8^+^ epithelial cells were nearly absent, confirming minimal epithelial contamination (Figure 2*B*). Clusters 2, 3, 5, and 6 expressed primary TNF receptors, whereas *Tnfrsf1a* expression was reduced in TNFR1-deficient cluster 1 (Figure 2*B*). TNFR1-deficient cluster 1 showed elevated extracellular matrix remodeling genes, including *Fbln5*, *Ccl7*, *Col12a1*, *Col5a1*, *Col4a1*, and *Col5a2*, whereas TNF signaling–associated genes enriched in control cluster 6, including *Jun*, *NfkBia*, *Nfkbb2*, *Mmp9*, *Tnfrsf9*, *Pdgfra*, and *Traf1*, were largely depleted (Figure 2*B*). Clusters 2 (control) and 4 (TNFR1-deficient) lacked distinct molecular signatures and primarily expressed general mesenchymal markers, including *Actb* and *Vim*; despite passing quality control, they were excluded from further analysis. Clusters 1, 3, 5, and 6 expressed colonic myofibroblast markers, including *Pdgfra*, *Gata6*, *Col1a1*, *Fn1*, *Wnt5a*, *Gli1*, and *Foxl1*, supporting the presence of distinct myofibroblast subpopulations. Notably, control cluster 6, defined by unique markers absent in TNFR1-deficient cultures, indicated a TNFR1-dependent mesenchymal population (Figure 2*B* and *C*). Overall, TNFR1 loss promoted a more homogeneous mesenchymal transcriptional state with altered pathway signatures.

**Figure 2:**
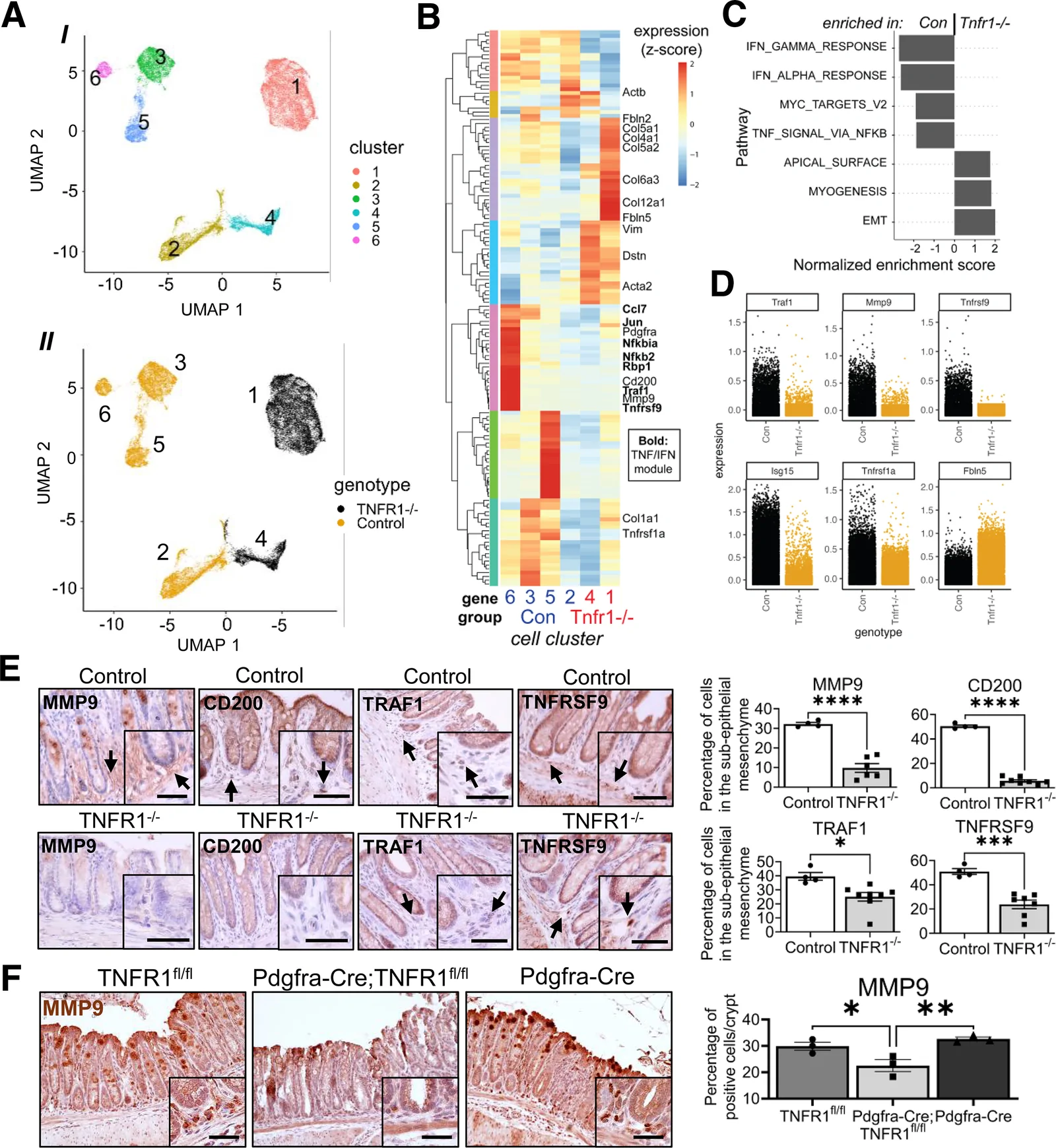
**TNFR1 is required to preserve colonic mesenchymal cell (MSC) diversity including a novel subpopulation defined by TNF and IFN signaling genes.** (*A*) (*I*) UMAP identifies 6 clusters in 34,415 colonic myofibroblasts derived from control (TNFR1^+/−^) and TNFR1^−/−^ mice, merged and clustered together after sequencing, reflecting completely individualistic clusters. (*II*) Distinct association of clusters 1 and 4 to the TNFR1^−/−^ colonic myofibroblasts and clusters 2, 3, 5, and 6 to the control colonic myofibroblasts genotype across 3 individual animals (n = 3) from each genotype. (*B*) Dot plot showing expression of significantly modified genes against detected clusters. (*C*) Expression of classical mesenchymal identity markers in control and TNFR1^−/−^ colonic myofibroblasts, segregated across the clusters. (*D*) Gene Ontology pathways showing significant enrichment among top marker genes for cluster 1 and 6 derived from control and TNFR1^−/−^ colonic myofibroblasts. (*E*) Immunostaining of MMP9, CD200, TNFRSF9, and TRAF1 highly enriched in cluster 6 from control colonic myofibroblasts and significantly diminished in cluster 1 of TNFR1^−/−^ colonic myofibroblasts, in native colon of control and TNFR1^−/−^ mice. (*F*) Immunolocalization and the percentage of MMP9-positive cells in the subepithelial and pericryptal niche of distal colon of 6-week-old Pdgfra-Cre;TNFR1^fl/fl^ mice, relative to TNFR1^fl/fl^ and Pdgfra-Cre controls. The *black dotted line* indicates the border of the subepithelial mesenchymal compartment used for analysis. *Black arrows* indicate positively stained cells. Each *dot* represents an individual biological replicate (number), with 15–20 crypts averaged from the distal colon adjacent to the anal region. Scale bars, 50 μm. All images are representative of n = 4–8 animals. Data were analyzed by an unpaired Student *t* test or a 1-way analysis of variance, where ∗*P* < .05; ∗∗∗*P* < .001, ∗∗∗∗*P* < .0001, and ^#^*P* = .05–.9.

To further define cluster relationships, we identified cluster-specific markers and the associated signaling pathways. TNFR1-deficient cluster 1 contained 190 markers, including *Meg3*, *Crlf1*, and *Fbln5*, linked to epithelial mesenchymal transition, myogenesis, UV response downregulation, and cGMP signaling (Figure 2*B* and *C*). Control cluster 3 contained only 18 markers, including shared expressions of *Col1a1* and *Fbln2* with clusters 1 and 6 (Figure 2*B* and *D*), preventing meaningful pathway enrichment analysis. Control cluster 5 was characterized by 293 markers, including *Smim1* and *Ctla2a*, but showed no significantly enriched pathways (q < 0.05). In contrast, cluster 6 expressed 190 unique genes, including *Mmp9*, *Cd200*, and *Traf1*, associated with transcriptional responses to TNF, interferon (IFN), and other cytokines (Figure 2*B*–*D*).

The loss of distinct cluster 6 markers in TNFR1^−/−^ colonic myofibroblasts, identified by Uniform Manifold Approximation and Projection (UMAP) and gene clustering in Figure 2*B* and *D*, was confirmed in TNFR1^−/−^ animals. These markers were selected based on their high magnitude of upregulation in cluster 6 of control colonic myofibroblasts, and their near-complete depletion in TNFR1^−/−^ colonic myofibroblasts. By immunostaining, there was a 70% reduction in MMP9+; 89% in CD200+; 37% in TRAF1+; and 53% in TNFRSF9+ cells in TNFR1^−/−^ animals relative to controls (Figure 2*E*). Consistently a reduction in MMP9+ subepithelial mesenchymal cells was seen in Pdgfra-Cre;TNFR1^fl/fl^ mice relative to TNFR1^fl/fl^ and Pdgfra-Cre controls (Figure 2*F*), demonstrating a specific role of pericryptal mesenchymal TNFR1 expression in the regulation of the cluster 6 cells. Thus, cluster 6 represents a specialized population of mesenchymal cells distinguished by enrichment of TNF signaling pathways. In the absence of TNFR1, this cluster fails to emerge, and the overall colonic myofibroblasts population is correspondingly less diverse. Thus, TNFR1 regulates the colonic homeostatic mesenchymal cell census and its diversification into specialized subpopulations. Importantly, TNFR1 on PDGFRα+ mesenchymal cells seem to determine the presence of subepithelial mesenchymal cells that are a hub for TNF, IFN, and other cytokine signaling.

### Transcriptome Profiling of Tumor Necrosis Factor Receptor 1–Deficient Colonic Myofibroblasts Identifies Upregulation of Integrin A6 but Loss of the Epithelial Stem Cell Niche Factor R-Spondin 3

Although single-cell experiments enable profiling of mesenchymal cell diversity, they can miss crucial gene products expressed at low levels. To distinguish TNFR1^−/−^ from control colonic myofibroblasts, we therefore investigated expression via bulk RNA sequencing (RNA-seq) analysis using cultured primary myofibroblasts. Bulk and single-cell transcriptome analyses of primary mouse TNFR1-depleted colonic myofibroblasts identified disruptions in cytokine signaling pathways across subepithelial mesenchymal clusters, consistent with patterns observed in human ulcerative colitis mesenchyme.^19^ A Kyoto Encyclopedia of Genes and Genomes pathway analysis of differentially expressed genes showed that the loss of TNFR1 alters many biologically significant processes including anchoring and cell substrate junctions, focal adhesions, extracellular matrix, and actin binding processes (Figure 3*A*). One gene relevant to enhanced matrix signaling, *Itga6*, was significantly upregulated in TNFR1^−/−^ compared with control colonic myofibroblasts (Figure 3*B, CI and II*). Furthermore, we found a specific reduction in key stem cell niche factors such as *Rspo3*, *Wnt2b*, and *Fgf2* in TNFR1-deficient colonic myofibroblasts (Figure 3*B*). Notably, *Rspo3*, crucial for leucine-rich repeat-containing G protein-coupled receptor 5 (Lgr5)+ stem cell maintenance,^22^^,^^23^ was downregulated 5.1-fold in TNFR1^−/−^ colonic myofibroblasts (Figure 3*B*). In vivo, we found that, in germline TNFR1-deficient mice (Figure 3*B*), similar to specific targeted deletion of TNFR1 in PDGFRA+ mouse cells (Pdgfra-Cre;TNFR1^fl/fl^ animals), R-spondin 3 (Rspo3) levels were reduced in the pericryptal mesenchyme (Figure 1*C III*). Thus, pericryptal mesenchymal TNFR1 regulates expression of the Rspo3 niche factor in a cell-autonomous manner.

**Figure 3:**
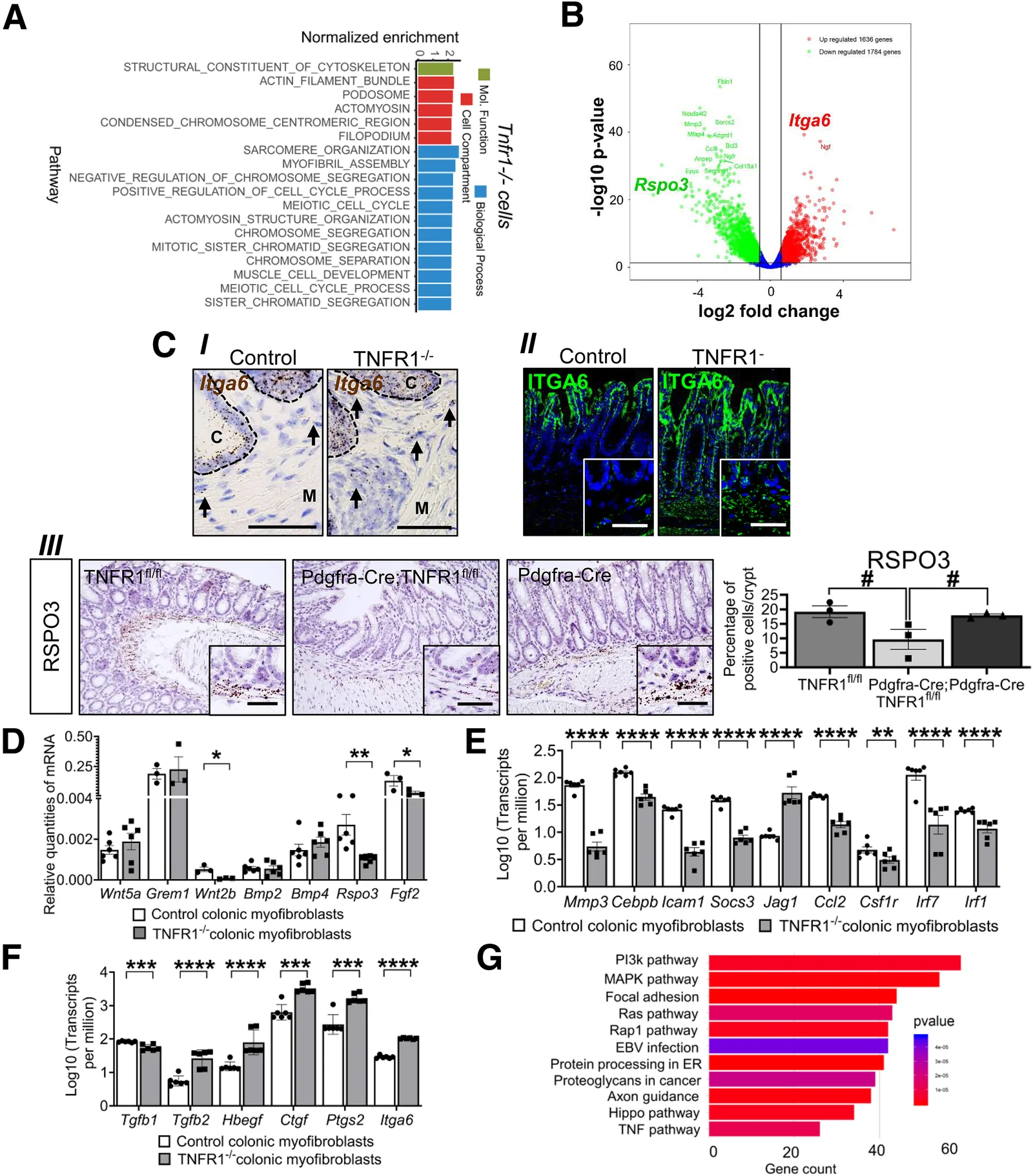
**Transcriptome profiling of TNFR1-deficient colonic myofibroblasts identifies upregulation of ITGA6 but loss of the epithelial stem cell niche factor R-spo3.** (*A*) Kyoto Encyclopedia of Genes and Genomes (KEGG) pathway analysis from RNA-seq showing biological pathways, cellular components, and molecular function of differently expressed genes (DEGs) in TNFR1^−/−^ vs control colonic myofibroblasts, as gene ratios. (*B*) A volcano plot demonstrating significantly modified DEGs. (*C*) Localization of ITGA6 in the distal colon of 16-week-old control and TNFR1^−/−^ mice by (*I*) in situ hybridization and (*II*) immunofluorescence staining. Subsets show the localization of ITGA6 in the pericryptal niche cells indicated by *black arrows* (C, crypts; M, mesenchyme). (*III*) Immunolocalization and the percentage of RSPO3-positive cells in the subepithelial and pericryptal niche of distal colon of 6-week-old Pdgfra-Cre;TNFR1^fl/fl^ mice, relative to TNFR1^fl/fl^ and Pdgfra-Cre controls. The *black dotted line* indicates the border of the subepithelial mesenchymal compartment used for analysis. Each *dot* represents an individual biological replicate (number), with 15–20 crypts averaged from the distal colon adjacent to the anal region. (*D*) Quantitative PCR analysis of Wnt and BMP ligands expressed in control and TNFR1^−/−^ colonic myofibroblasts, at baseline. Genes associated with (*E*) wound healing and mucosal repair and (*F*) TNF signaling as determined by RNA-seq of TNFR1^−/−^ vs control colonic myofibroblasts, expressed in transcripts per million. (*G*) Gene Ontology annotation of the top pathways with DEGs sorted by *P* value of TNFR1^−/−^ vs control colonic myofibroblasts. Scale bars, 50 μm. All images are representative of n = 3 animals and were taken using a Zeiss LSM 710 Inverted Confocal Microscope. Error bars are presented as mean ± standard error of the mean. Data are analyzed by an unpaired Student *t* test or a 1-way analysis of variance, where ∗*P* < .05, ∗∗*P* < .01, ∗∗∗*P* < .001, and ∗∗∗∗*P* < .0001.

Interestingly, in contrast to the shifts in basal crypt stem cell niche factors, no major differences were observed in the transcript levels of “upper crypt” (ie, differentiated) niche factors^24^ such as *Wnt5a*, *Bmp2*, and *Bmp4* (Figure 3*D*). Several transcripts or pathways associated with wound healing, including *Tgfb2*, *Hbegf*, *Ctgf*, *Ptgs2*, *Itga6,* and *Il6,* and with proliferation and differentiation (eg, phosphoinositide 3-kinase [PI3K], mitogen-activated protein kinase [MAPK], Ras-associated protein 1 [RAP1], transforming growth factor β, and RAS) were upregulated in TNFR1^−/−^ colonic myofibroblasts, whereas TNF signaling genes associated with immunity to microbial infections were downregulated (*Mmp3*, *Cebpb*, *Icam1*, *Socs3*)^13^ (Figure 3*E*–*G*). Moreover, we observed upregulation of *Jag1* and several other genes (eg, *Ccl2*, *Csf1r*, *Irf1* and *Irf7*) involved in mucosal repair and in adaptive immune response (Figure 3*F*). Thus, deletion of TNFR1 from colonic myofibroblasts alters mesenchymal cell–matrix interactions while downregulating stem cell–associated niche transcript expression and mucosal immunity.

### Loss of Tumor Necrosis Factor Receptor 1 in Platelet-Derived Growth Factor Receptor α^+^ Mesenchymal Cells Impairs Intestinal Stem Cell Expression and Epithelial Proliferation

We have shown previously that targeted TNFR1 deletion in the intestinal mucosa results in an altered colonic mucosal phenotype with increased neutrophil accumulation, decreased epithelial proliferation, increased DNA damage, and crypt abscess.^11^ Here, we show that the TNFR1-deletion phenotype relative to TNFR1^+/−^ controls is also characterized by alterations in mesenchymal cell expression patterns. Selective deletion of TNFR1 in the PDGFRα+ mesenchymal cell lineage recapitulates increased neutrophil accumulation, decreased epithelial proliferation, and increased DNA damage in Pdgfra-Cre;TNFR1^fl/fl^ animals relative to TNFR1^fl/fl^ and Pdgfra-Cre controls (Figure 3*A*), Thus, cell-specific TNFR1 deletion in PDGFRα+ cells in vivo selectively alters the colonic mesenchymal cell census.

Because epithelial proliferation is maintained by stromal support of intestinal stem cells, we next investigated whether the proliferation defect in Pdgfra-Cre;TNFR1^fl/fl^ mice was associated with impaired stem cell maintenance. Pdgfra-Cre;TNFR1^fl/fl^ animals demonstrated a significant decrease in Lgr5+ (Figure 4*BI*) and Ephb3+ (Figure 4*BII*) stem cells. Similarly, we compared the colons of TNFR1^−/−^ mice with their heterozygote littermate controls (TNFR1^+/−^). Consistently, there was a 44% reduction in Lgr5+ stem numbers and a ∼2-fold decrease in Lgr5 messenger RNA (mRNA) expression (Figure 4*CI*) in TNFR1^−/−^ animals. Similar results were obtained from an analysis of *Ephb3* expression (Figure 4*CII*). Together, these findings demonstrate that TNFR1 signaling in PDGFRα^+^ mesenchymal cells is required to maintain intestinal stem cell expression. The loss of TNFR1 leads to reduced Lgr5^+^ and Ephb3^+^ stem cells, consistent with decreased epithelial proliferation, indicating that disrupted mesenchymal TNFR1 signaling impairs stem cell maintenance and epithelial regeneration in vivo.

**Figure 4:**
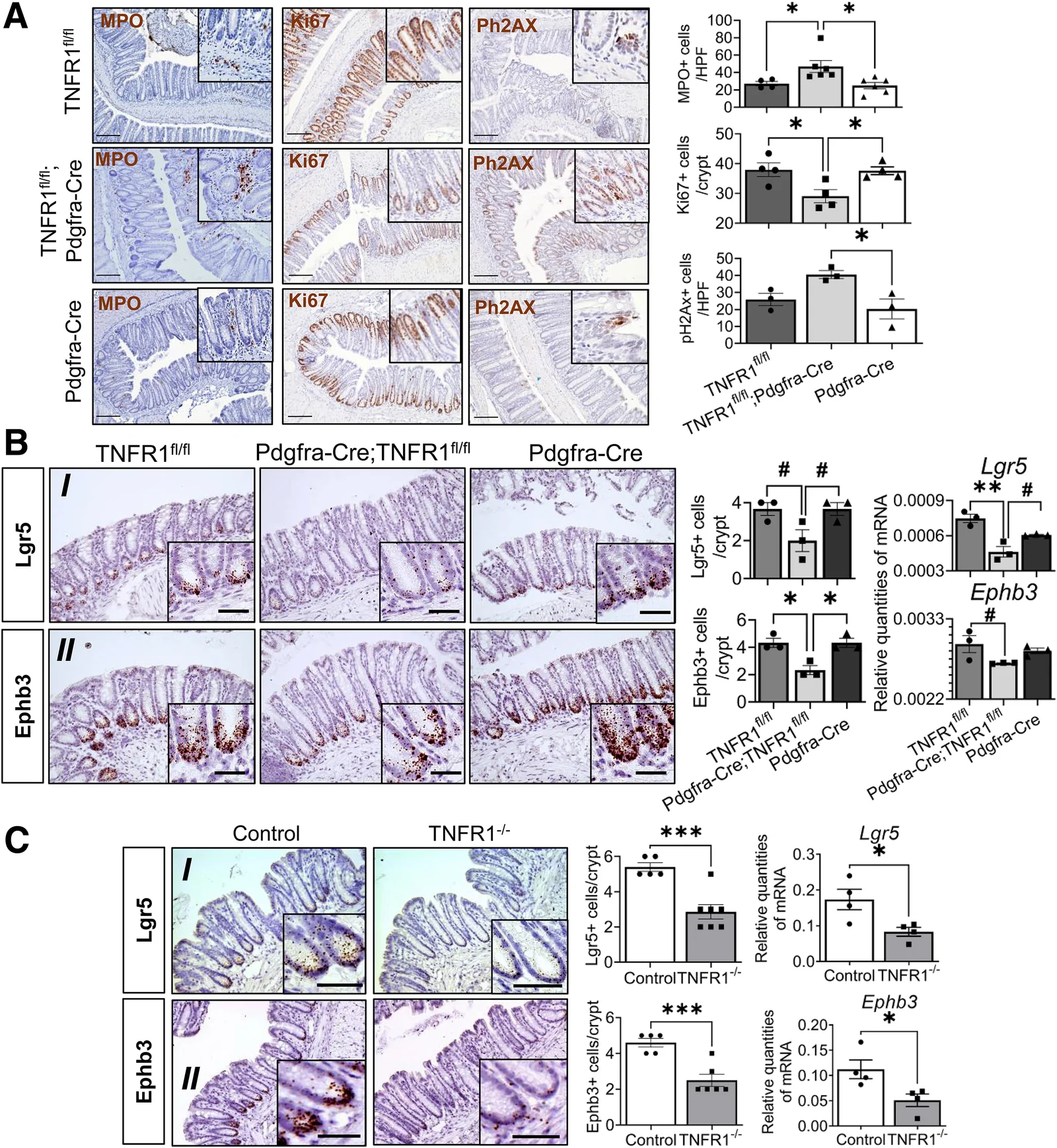
**TNFR1 deletion in colonic pericryptal PDGFRα+ cells results in a loss of proliferation and stem cell marker expression.** Immunolocalization and quantification of (*A*) MPO+ cells, Ki67+, and Ph2AX+ cells in the distal colon of Pdgfra-Cre;TNFR1^fl/fl^ mice, relative to TNFR1^fl/fl^, and Pdgfra-Cre control animals (8 weeks) through in situ hybridization. Quantification of MPO+, Ph2AX+, or Ki67+ cells were carried out per high powered field (HFP) in crypt epithelial cells and in the subepithelial mesenchyme. Scale bars, 200 μm. (*B*) Localization and quantification of (*I*) Lgr5+ cells per crypt and quantitative PCR (qPCR) analysis of *Lgr5* expression, or (*II*) Ephb3+ cells per crypt and qPCR analysis of *Ephb3* expression from the distal colon of TNFR1^fl/fl^, Vil1-Cre;TNFR1^fl/fl^, and LysM-Cre;TNFR1^fl/fl^ mice. (*C*) Localization and quantification of (*I*) Lgr5+ cells per crypt and qPCR analysis of *Lgr5* expression, or (*II*) Ephb3+ cells per crypt and qPCR analysis of *Ephb3* expression from the distal colon of control (TNFR1^+/−^) and TNFR1^−/−^ mice. Subsets show the punctate staining of Lgr5 and Ephb3 in the crypt base cells. Each *dot* represents an individual biological replicate (number), with 15–20 crypts averaged from the distal colon adjacent to the anal region. Scale bars, 50 μm. All images are representative of n = 3–8 animals. Error bars are presented as mean ± standard error of the mean. Data are analyzed by an unpaired Student *t* test or a 1-way analysis of variance, where ∗*P* < .05, ∗∗*P* < .01, ∗∗∗*P* < .001, and ^#^*P* = .05–.09.

### Tumor Necrosis Factor Receptor 1–Dependent R-Spondin 3 Expression in Mesenchymal Cells Sustains the Leucine-Rich Repeat-Containing G Protein-Coupled Receptor 5+ Intestinal Stem Cell Compartment

To determine the functional consequences of reduced intestinal stem cell expression and epithelial proliferation in vivo, and decreased expression of key niche factors including RSPO3 in TNFR1^−/−^ colonic myofibroblasts, we established an epithelial-mesenchymal co-culture model. By coculture of control or TNFR1^−/−^ colonoids with control or TNFR1^−/−^ mesenchymal cells in a 3-dimensional matrix (Figure 5*A*), we studied the effects of a specific genotype on epithelial and mesenchymal expression and function. Coculture of control or TNFR1^−/−^ colonoids with TNFR1^−/−^ colonic myofibroblasts significantly reduced total mRNA expression of both *Lgr5* and *Ephb3* (an alternate stem cell marker^25^^,^^26^) by 2-fold (*P* = .02), compared with coculture of control or TNFR1^−/−^ organoids with control colonic myofibroblasts (Figure 5*BI*). Moreover, coculture with TNFR1^−/−^ colonic myofibroblasts altered the morphology of colonoids, resulting in a 40% decrease in the proportion of budding colonoid structures,^25^ with a parallel increase in a rapidly expanding monolayer-like epithelium (Figure 5*BII*). Thus, mesenchymal TNFR1 regulates the epithelial stem cell compartment and functionally alters the growth pattern of colonoids.

**Figure 5:**
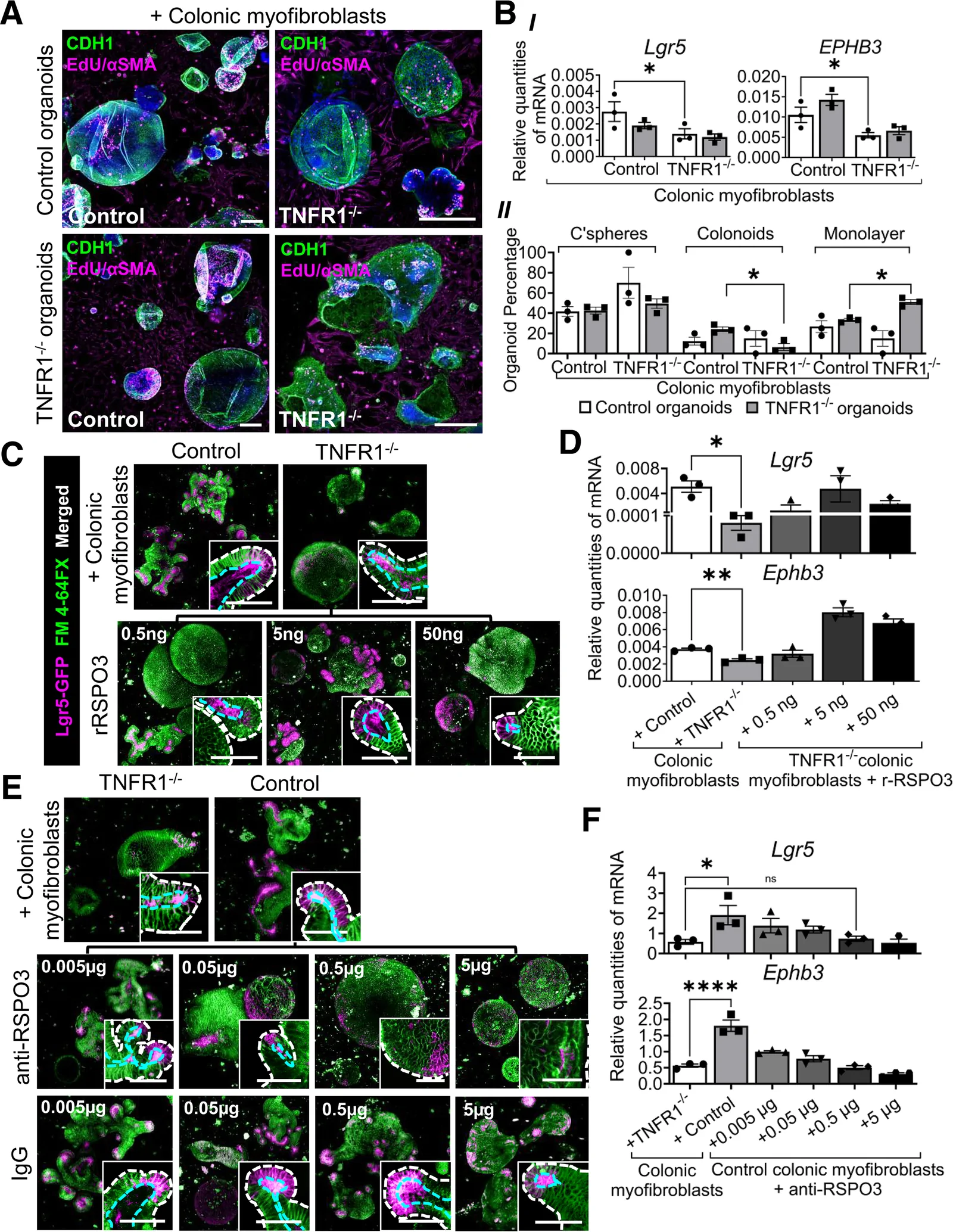
**RSPO3 expression by mesenchymal cells is required for homeostatic expression of stem cell numbers and function in TNFR1^−/−^ epithelium.** (*A*) Immunofluorescence staining of epithelial E-cadherin (CDH1); 5-ethynyl-2′-deoxyuridine (EdU)+ and aSMA+ colonic myofibroblasts in cocultures of control or TNFR1^−/−^ organoids with control or TNFR1^−/−^ colonic myofibroblasts. (*B*) Quantitative PCR (qPCR) analysis of (*I*) *Lgr5* and *Ephb3* mRNA expression, and (*II*) quantification of colonospheres, colonoids and colonoid monolayers, in cocultures of control or TNFR1^−/−^ organoids with control or TNFR1^−/−^ colonic myofibroblasts. (*C*) Immunofluorescence staining of green fluorescent protein (GFP) and FM 4-64X, and (*D*) q-PCR analysis of *Lgr5* and *Ephb3* levels in Lgr5-GFP organoids cocultured with untreated control or TNFR1^−/−^ colonic myofibroblasts and TNFR1^−/−^ colonic myofibroblasts treated with increasing concentrations of r-RSPO3 (0.5–50 ng/mL) for 5 days in culture. (*E*) Immunofluorescence staining of GFP and FM 4-64X, and (*F*) qPCR analysis of *Lgr5* and *Ephb3* levels in Lgr5-GFP organoids cocultured with untreated TNFR1^−/−^ or controls colonic myofibroblasts and controls colonic myofibroblasts treated with increasing concentrations of anti-RSPO3 antibody and its IgG isotype control (0.005–5 μg/mL) for 5 days in culture. The *cyan dotted line* represents the cell apical border, and the *white line* denotes the basal border. Scale bars, 50 μm. All images are representative of n = 3 animals and were taken using a Zeiss LSM 710 Inverted Confocal Microscope. Each *dot* represents an individual biological replicate (number), with 5–15 organoids averaged from each animal. Error bars are presented as mean ± standard error of the mean. Data are analyzed by a 1-way analysis of variance, where ∗*P* < .05, ∗∗*P* < .01, and ∗∗∗∗*P* < .0001.

We next asked whether RSPO3 was sufficient to restore functional support of Lgr5+ epithelial cells in the presence of TNFR1^−/−^ colonic myofibroblasts. Colonoids from Lgr5::DTR-EGFP mice were cocultured with TNFR1^−/−^ colonic myofibroblasts supplemented with increasing concentrations of recombinant RSPO3 (r-RSPO3) for 5 days. Colonoids treated with 5 ng r-RSPO3 showed visibly more crypt-like budding structures compared with untreated controls (Figure 5*C*) and restored expression to *Lgr5* and *Ephb3* to control levels (Figure 5*D*). However, treatment with higher RSPO3 concentrations (50 ng) reduced the number of crypt-like budding structures, with Lgr5–green fluorescent protein expression becoming diffusely distributed throughout the organoid rather than concentrated in budding regions. Concurrently, the levels of *Lgr5* and *Ephb3* were also reduced in these colonoids. To confirm the specificity of r-RSPO3 in restoring stem cell marker expression, control colonic myofibroblasts were treated with an anti-RSPO3 antibody, and stem cell expression in organoid cocultures was measured. Anti-RSPO3 inhibited *Lgr5* and *Ephb3* expression in a dose-dependent manner, such that at the 0.05-μg dose, the levels of *Lgr5* and *Ephb3* expression are comparable to those found in cocultures with TNFR1^−/−^ colonic myofibroblasts (Figure 5*E and F*). Therefore, RSPO3 rescues the loss of mesenchymal TNFR1 signaling by maintaining epithelial stem cells, preserving their marker expression and functional activity.

### Inhibiting Integrin A6 Rescues Functional Defects in Proliferation, Wound Healing, and Stem Cell Niche Factor Expression in Tumor Necrosis Factor Receptor 1^−/−^ Colonic Myofibroblasts

To investigate the roles of the proliferation-associated pathways, PI3K, MAPK, and RAP1 identified in our transcriptome analysis (Figure 3*F*), in mesenchymal proliferation and wound healing, we analyzed these functional states in control and TNFR1-deficient primary colonic myofibroblast cultures. Immunoblotting showed that TNFR1^−/−^ colonic myofibroblasts had elevated baseline PI3K and MAPK signaling, with phosphorylated AKT and extracellular signal–regulated kinase (ERK) levels increased approximately 4-fold compared with untreated controls (Figure 6*A*). RAP1–GTPase-activating protein was also upregulated ∼2-fold in TNFR1^−/−^ colonic myofibroblasts (Figure 6*A*). Functionally, TNFR1^−/−^ colonic myofibroblasts migrated more rapidly in a 2-dimensional scratch wound assay (Figure 4*B*), demonstrating that TNFR1 limits overall mesenchymal proliferation–associated pathways and migration but may selectively maintain the PDGFRα+ pericryptal subpopulation, highlighting pathway-specific regulation of distinct colonic myofibroblasts subsets.

**Figure 6:**
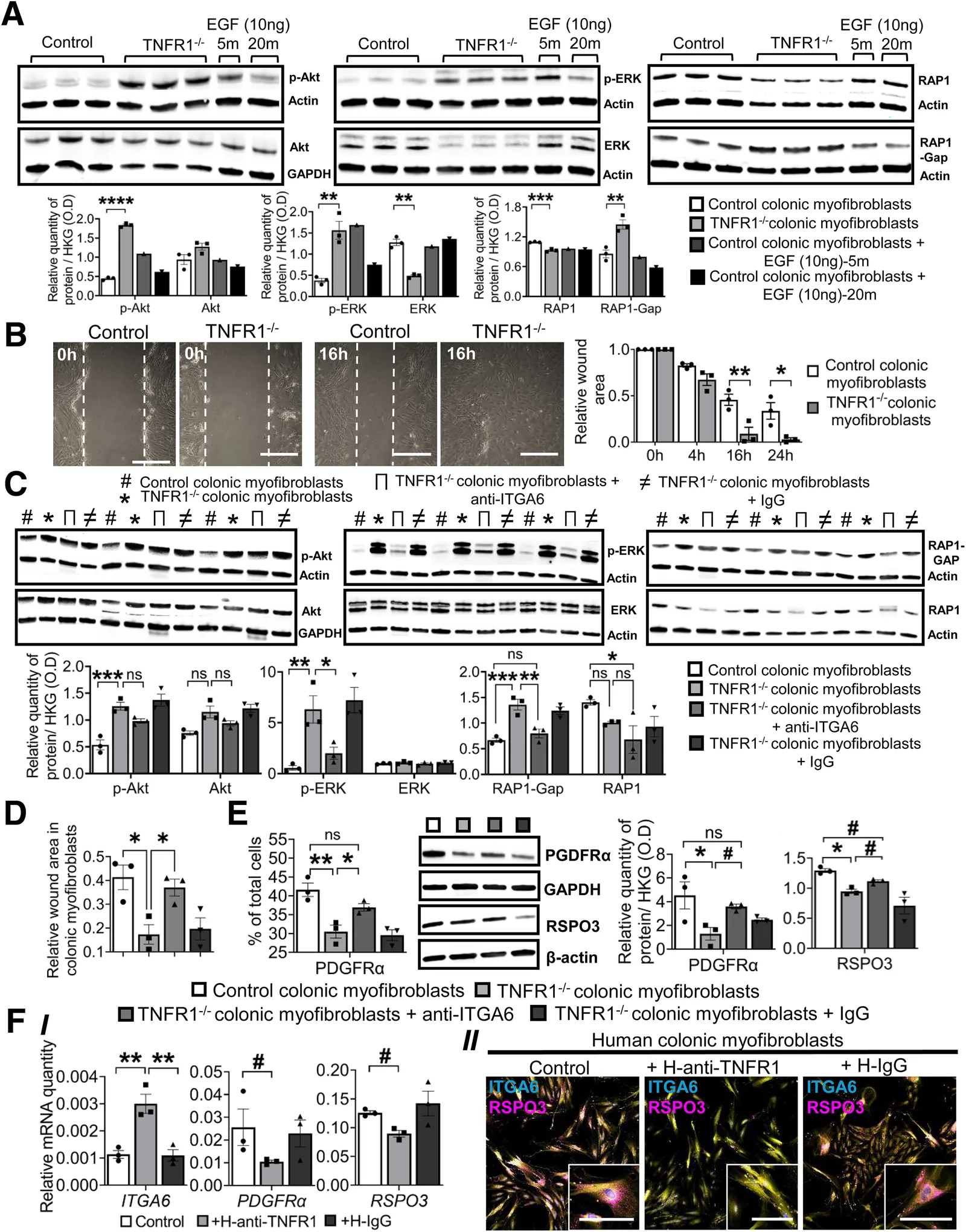
**Inhibiting ITGA6 rescues functional defects in proliferation, wound healing, and stem cell niche factor expression in TNFR1^−/−^ colonic myofibroblasts.** (*A*) Activation of the PI3K, MAPK, and RAP1 pathways in whole-cell lysates of untreated control and TNFR1^−/−^ colonic myofibroblasts, determined by immunoblotting. Positive controls included control colonic myofibroblasts treated with 10 ng/mL epidermal growth factor (EGF) for 5 or 20 minutes. Total protein levels of p-Akt (Ser 473), Akt; p-ERK (Thr202/Tyr204), ERK; and RAP1–GTPase-activating protein (GAP), RAP1 was quantified relative to housekeeping genes, Actin, or GAPDH. (*B*) Cell migration as determined by wound scratch assays of untreated control and TNFR1^−/−^ colonic myofibroblasts at baseline, over 0–24 hours. Migrated cells were quantified by determining the wound closure rate relative to time = 0. Scale bars, 50 μm. Phase contrast images were taken using a Leica DM IL LED inverted microscope (Leica) equipped with a 5×/0.12 NA objective and a digital camera. (*C*) Activation of the PI3K, MAPK, and RAP1 pathways in whole-cell lysates of TNFR1^−/−^ colonic myofibroblasts treated with 0.2 μg anti-ITGA6 antibody and IgG control, relative to untreated control colonic myofibroblasts, determined by immunoblotting. The total protein levels of p-Akt, Akt; p-ERK, ERK, and RAP1-GAP, RAP1 were quantified relative to housekeeping genes, actin, or GAPDH. n = 3 animals. (*D*) Cell migration determined by wound-scratch assays (over 0–16 hours) and quantification of pericryptal PDGFRα-positive cells in TNFR1^−/−^ colonic myofibroblasts treated with 0.2 μg anti-ITGA6 antibody and IgG control, relative to untreated control colonic myofibroblasts. (*E*) Quantification of total protein levels of PDGFRα and RSPO3 in TNFR1^−/−^ colonic myofibroblasts treated with 0.2 μg anti-ITGA6 antibody and IgG control, relative to untreated control colonic myofibroblasts. (*F*) (*I*) qPCR analysis of *ITGA6*, *PDGFRα*, and *RSPO3*, and (*II*) localization of ITGA6 and RSPO3 in primary human colonic myofibroblasts, 18Co, treated with 0.5 μg/mL anti-TNFR1 and its IgG isotype control for 5 days, imaged using a Zeiss LSM 710 inverted confocal microscope. Scale bars, 25 μm. All images are representative of n = 3 animals. Error bars are presented as mean ± standard error of the mean. Data are analyzed by a 1-way analysis of variance, where ∗*P* < .05, ∗∗*P* < .01, ∗∗∗*P* < .001, and ^#^*P* = .05–.09.

Integrin α6 (ITGA6) is a laminin-binding adhesion receptor expressed on colonic mesenchymal and stem-like cells, where it regulates adhesion, migration, and proliferation^27^; given these functions, we next investigated the role of ITGA6 in controlling proliferation and migration of TNFR1^−/−^ colonic myofibroblasts. TNFR1^−/−^ colonic myofibroblasts were treated with an anti-ITGA6 blocking antibody to determine if it rescued its altered proliferation-associated pathways and migratory capacity. Blocking ITGA6 restored ERK and RAP1 signaling (Figure 6*C*), and normalized wound closure rates (Figure 6*D*), demonstrating that ITGA6 mediates the enhanced proliferative and migratory phenotype of TNFR1^−/−^ colonic myofibroblasts. Because the crosstalk between integrins and PDGFRs is essential to effective growth factor signaling,^27^ we next assessed ITGA6’s role in stem cell niche function. Anti-ITGA6 antibody treatment of TNFR1^−/−^ colonic myofibroblasts restored the numbers of PDGFRα+ cells, expression of PDGFRα protein, and expression of RSPO3 to near control levels (Figure 6*E*). These results indicate that elevated ITGA6 in TNFR1^−/−^ colonic myofibroblasts contributes to their enhanced proliferation, migration, and maintenance of PDGFRα+ cells, highlighting ITGA6 as a key regulator of mesenchymal function in the absence of TNFR1.

To determine whether these findings translate to the human intestinal mesenchyme, we treated human primary 18Co myofibroblasts with 0.5 μg of anti-TNFR1 antibody to block receptor signaling. Treated cells exhibited significant increase in the transcript levels of *ITGA6* and a downregulation of *PDGFRA* and *RSPO3* (Figure 6*F I*), similar to mouse TNFR1^−/−^ colonic myofibroblasts. Furthermore, we showed that ITGA6 and RSPO3 colocalize in human primary 18Co myofibroblasts and that treatment of these cells with the anti-TNFR1 antibody reduced the balance of RSPO3/ITGA6 expression (Figure 6*F II*). Therefore, TNFR1 regulates ITGA6 and the stem cell niche factors, PDGFRα and RSPO3, in both mouse and human colonic mesenchymal cells. Hence, in summary, we show that mesenchymal TNFR1 is required for preserving colonic mesenchymal diversity, regulating RSPO3 dependent pericryptal niche signaling, and maintaining the intestinal stem cell compartment. Loss of TNFR1 disrupts mesenchymal heterogeneity, compromises stem cell support, and is partially mediated through ITGA6-associated dysregulation.

## Discussion

The profusion of therapies that inhibit TNF signaling in the context of autoimmunity necessitates a fuller understanding of their potential physiological impact. This is a critical need due to the high rates of clinical nonresponse and loss of response in IBD.^4^ Studies have demonstrated protective and physiological roles of TNF in IBD^8^, ^9^, ^10^, ^11^ and reported new-onset IBD with the use of anti-TNF agents,^28^ consistent with possible anti-inflammatory and/or reparative roles for TNF in the intestine. Although previous studies have supported a role for TNF signaling in the maintenance of the epithelium and healing responses in murine intestinal inflammation models, the mechanisms have remained unknown and have been thought to be epithelial cell–autonomous.^8^, ^9^, ^10^, ^11^ Our study shows that TNFR1-mediated signaling is central to the proper specification of colonic pericryptal mesenchymal cells and to the fundamental mesenchymal–epithelial paracrine axis that maintains the crypt stem cell pool. This cytokine signal is essential in shaping mesenchymal cellular diversity of the colon. Combined with recent work showing TNFR1’s role in mediating mucosal tolerance to the microbiome in preclinical IBD models,^11^ our study outlines a multimodal mechanism, with mesenchymal cells regulating the epithelial response to cytokines, through which TNFR1 signaling supports mucosal homeostasis. In this study, the genetic or antibody-mediated depletion of TNFR1 signaling had profound effects on mesenchymal cell function and epithelial stem cell census, key aspects of mucosal health. Future studies should explore how these intercellular networks are altered in patients treated with anti-TNF agents.

We report that mesenchymal TNFR1 is required to preserve the overall diversity of colonic mesenchymal populations at homeostasis, including a subpopulation of pericryptal cells that are uniquely enriched for TNF and IFN signaling genes. TNFR1 ablation in vivo alters the proportions of mesenchymal cell subpopulations. As there are several known markers of mesenchymal subpopulations with potential overlap, we performed single-cell RNA transcriptomics to precisely enumerate the diversity of colonic mesenchymal cell types. Moreover, as the “cytokine storm” of IBD is associated with broad changes in mesenchymal cell differentiation,^15^^,^^19^, ^20^, ^21^ we had anticipated that synergy between many cytokines could drive these changes. However, in single-cell experiments, we found that the deletion of a single cytokine receptor results in fundamental changes such that TNFR1^−/−^ and TNFR1^+/−^ cell clusters were mostly mutually exclusive. Although cluster exclusion can sometimes arise from batch effects, these analyses were performed as a single batch with independent cultures from cohoused littermate mice. Thus, we believe the results are faithful to the composition of the cultures; however, we cannot rule out elimination or modification, due to culturing, of certain cell types initially present in vivo. However, our analysis, on several occasions, demonstrates that in vitro cultures faithfully recapitulated the alterations in the major mesenchymal phenotypes observed in vivo, and this was further validated by both scRNA-seq and bulk RNA-seq data. Multiple approaches confirmed that a mesenchymal cell type appears to be downregulated in TNFR1^−/−^ mice. This cell type is located near the base of the crypt, expresses Mmp9 and Cd200 markers, and has a transcriptional profile with hallmarks of TNF signaling. The cell type appears to be a subset of PDGFRα+ cells and shares some similarity with an ulcerative colitis–associated mesenchymal cell type.^15^ Future studies will need to characterize how this cell type integrates with other colonic myofibroblast subpopulations in terms of hierarchy and niche function, as well as determine whether it has specific functions that distinguish it from other PDGFRα+ cells. Taken together, these results demonstrate that TNF is an essential driver of colonic myofibroblast specification and function.

Our study defines a novel pathway of TNFR1-driven repression of integrins (ITGA6), the result of which is differential alteration of cell proliferation and wound healing-associated pathways within the mesenchymal subpopulations. Moreover, we found that the TNFR1^−/−^ colonic myofibroblasts upregulating ITGA6 fail to perform 1 of their key functions: the secretion of sufficient niche-specific growth factors to sustain the epithelial stem cell pool.^27^ The effects of TNFR1 signaling on the stem cell niche converge on RSPO3 expression. Supplementation of TNFR1^−/−^ colonic myofibroblasts with exogenous RSPO3 in vitro restored the number of Lgr5+ stem cells to control levels. RSPO3, the most highly expressed intestinal Wnt ligand, is crucial for sustaining the Lgr5+ stem cell pool and is thought to be entirely produced by pericryptal niche cells.^22^^,^^23^^,^^29^ Consistent with our findings, PDGFRα+ pericryptal mesenchymal cells have been identified as a key source of RSPO3^23^ and support epithelial architecture and the renewal of stem cells during both homeostasis and the injury response.^30^^,^^31^ Hence, the development of approaches to restore RSPO3-mediated signaling or inhibit ITGA6 may partially compensate for the effects of TNFR1 ablation or anti-TNF on mesenchymal–epithelial interactions and the stem cell niche.

The deletion of mesenchymal TNFR1 has profound effects on epithelial stem cells in vivo and in vitro. We show in Figure 4 that animals with global and mesenchymal-specific TNFR1 depletion exhibit a loss of Lgr5+ stem cells. That this occurs without attendant changes in crypt height suggests the presence of other homeostatic mechanisms to maintain the overall crypt surface area. The coordinated mechanism through which mesenchymal TNFR1 regulates the epithelium through the niche factor expression reinforces the need for cell culture systems that partially reconstitute these heterotypic cellular interactions. We believe the epithelium and mesenchyme coculture model demonstrated here is a good example of this. Coculture systems will become increasingly important for investigation of cytokine signaling pathways, as these components are expressed by multiple tissue types in the mucosa, and for resolution of the mechanistic basis of inflammatory diseases. Our findings also provide insight into the limitations of current anti-TNF therapies by showing that TNFR1 signaling in colonic mesenchymal cells is not only involved in inflammatory signaling but is also required to maintain mesenchymal diversity, RSPO3-dependent stem cell niche function, and epithelial homeostasis. Therefore, the broad inhibition of TNF/TNFR1 pathways may unintentionally disrupt protective mesenchymal-dependent repair pathways that are important for maintaining intestinal regeneration and mucosal healing.

## Methods and Materials

All authors had access to the study data and reviewed and approved the final manuscript.

### Experimental Animals

The animals were cared for as per Institutional Animal Care and Use Committee rules and regulations at Children’s Hospital Los Angeles under internal protocol number 288 and University of California San Diego, S21028. Preceding dissection, the animals were humanely anesthetized with isoflurane and euthanized by cervical dislocation. C57BL/6J-WT (cat# 000664), TNFR1^−/−^ (cat# 002818), and Pdgfra-Cre (cat# 013148) mice were obtained from Jackson Laboratory. Lgr5::EGFP-IRES-CreERT2 (Fred de Sauvage, Genentech), and TNFR1^fl/fl^ (George Kollias, Alexander Fleming Biomedical Sciences Research Center) mice were kindly gifted. Mesenchymal Pdgfra-Cre mice were crossed to TNFR1^fl/fl^ mice to generate Pdgfra-Cre;TNFR1^fl/fl^ animals.^32^ TNFR1 deletion in mesenchymal PDGFRα+ cells were confirmed by genotyping from 3 independent Pdgfra-Cre;TNFR1^fl/fl^, TNFR1^fl/fl^, and Pdgfra-Cre animals. To maintain proper experimental controls, littermate controls of transgenic mice were cohoused for >4 weeks prior to experimental collection and analysis. Adult male and female mice (6 at 12–16 weeks) were involved in the study.

### Mice Tissue Collection

The colon and cecum were dissected from euthanized mice and cleared of fecal contents. For histology, the colon was Swiss-rolled and fixed in 10% neutral buffered formalin. Five-micrometer slices of the tissue were sectioned. The distal end of the colon (2 cm from the anus) was longitudinally split into half and processed for RNA and protein isolation and in the same manner for generation of organoids and primary mesenchymal cells.

### Murine Three-Dimensional Organoid Culture

The distal colonic tissues isolated from control (TNFR1^+/−^), TNFR1^−/−^, or Lgr5::EGFP-IRES-CreERT2 mice were processed for digestion and crypt isolation as per previously established protocols.^11^^,^^33^ For passage, the organoids were broken mechanically by syringe disruption using a 27 G × 1/2 needle (Becton Dickinson, cat# 305109), then transferred to fresh Matrigel. The passage was performed every 5–8 days with a 1:4 split ratio, and the medium was changed after every 2 to 3 days. Each experiment was repeated at least 3 times. Each condition was examined in triplicate with multiple (>30–50) organoids in each sample.

### Coculture Experiments

#### Isolation of primary colonic myofibroblasts

Primary mouse colonic myofibroblasts were isolated and maintained as described previously.^16^ Briefly, the primary colonic myofibroblasts isolated from the distal end of 16-week-old control (TNFR1^+/−^) and TNFR1^−/−^ mice were repeatedly stirred at 250 rpm in 5 mM EDTA–Hank’s Balanced Salt Solution medium (without Ca^++^ and Mg^++^) at 37 °C for 15 minutes for a total of 5 washes. Following 2 washes of ice-cold phosphate-buffered saline (PBS), the tissue was digested in Dulbecco’s Modified Eagle Medium (DMEM), low glucose, GlutaMAX Supplement, pyruvate (Gibco, cat# 10567014) plus penicillin–streptomycin (10,000 U/mL) with 0.31 mg/mL Dispase (Gibco, cat# 17105041) and 0.25 mg/mL collagenase Type XI (Gibco, cat# 17104019) for up to 60 minutes at 250 rpm at 37 °C, until the tissue appeared stringy. The digested tissue was treated with ACK lysis buffer (4.15 g NH_4_Cl, 0.5 g KHCO_3_, 18.6 mg Na_2_EDTA, 400 ml H_2_O, pH 7.2-7.4) for 5 minutes and then passed through a 100-μm cell strainer into 6-well dishes of colonic myofibroblasts growth medium. The primary cultures were maintained in a colonic myofibroblasts growth medium comprised of DMEM, low glucose, GlutaMAX Supplement, pyruvate (Gibco, cat# 10567014) plus 10,000 U/mL penicillin–streptomycin (cat# 15140163) with 10% fetal bovine serum (FBS; Corning, cat# 35-010-CV) and 1X recombinant human insulin, human transferrin, and sodium selenite solution (Sigma, cat# I3146), and 20 ng/mL recombinant murine epidermal growth factor (Peprotech, cat# 315-09) at 37 °C in a 5% CO_2_/air mix. After a 24-hour incubation, the nonadherent cells were washed away. The colonic myofibroblasts were grown in a 2-dimensional culture for amplification of the cells for the main experimental setup. The control or TNFR1^−/−^ primary colonic myofibroblasts were seeded at a density of 1 × 10^3^ cells/mL in parallel in 6-well plates and were grown to 70% confluence. At day 5, the colonic myofibroblasts in 2-dimensional culture recovered similar numbers of mesenchymal populations (VIM+, αSMA+, PDGFRα+, GLI1+, or FOXL1+) as the colonic myofibroblasts in 3-dimensional culture.

#### Tumor necrosis factor treatment of primary colonic myofibroblasts

Control or TNFR1^−/−^ primary colonic myofibroblasts were seeded at a density of 1 × 10^3^ cells/mL in parallel in 6-well plates and were grown to 70% confluence. The cells were starved (devoid of FBS) for 24 hours in DMEM, low glucose, GlutaMAX Supplement, pyruvate medium plus 10,000 U/mL penicillin–streptomycin, before treatment with 1 ng, 10 ng, or 100 ng/mL TNF (R&D Systems, cat# 410-MT) for 20 minutes. The primary colonic myofibroblasts were collected for RNA or protein isolation thereafter.

#### Organoid and myofibroblasts coculture

Briefly, the passaged organoids were broken into single cells mechanically by passing through a syringe and needle and were mixed with the colonic myofibroblasts at a ratio of 10:1 into Matrigel in a 24-well plate. The cocultures were grown and maintained in 60% IntestiCult (STEMCELL, cat# 06005) and 40% colonic myofibroblasts growth medium at 37 °C. The identity and purity of the isolated primary colonic myofibroblasts was determined by immunofluorescence for different myofibroblast cell populations.

#### Genotypic recombination in coculture experiments

The effects of the TNFR1^−/−^ mesenchymal genotype on the epithelium were determined by a series of recombination coculture experiments in which control or TNFR1^−/−^ organoids were cocultured with control or TNFR1^−/−^ colonic myofibroblasts for 5 days as described above.

#### Coculture experiments with recombinant R-spondin 3 and anti–R-spondin 3 treatments

The Lgr5::EGFP-IRES-CreERT2 organoids cocultured with TNFR1^−/−^ colonic myofibroblasts were supplemented with increasing doses (5–50 ng/mL) of r-Rspo3 (R&D Systems, cat# 4120-RS) at beginning of the passage for 5 days to determine its effect on stem cells, relative to Lgr5::EGFP-IRES-CreERT2 organoids cocultured with control colonic myofibroblasts. Similarly, the Lgr5::EGFP-IRES-CreERT2 organoids cocultured with control colonic myofibroblasts were treated with an anti-RSPO3 antibody (R&D Systems, cat# AF35001) or its IgG isotype control (R&D Systems, cat# 5-001-A) in increasing doses of 0.005 to 5 μg at passage for 5 days to investigate its inhibition of stem cells relative to Lgr5::EGFP-IRES-CreERT2 organoids cocultured TNFR1^−/−^ colonic myofibroblasts. The cocultures were incubated for 10 minutes with 2 mg/mL FM4-64X (Thermo Fisher Scientific, cat# F34653), were diluted in PBS, and were subsequently imaged using a Zeiss LSM 710 Inverted Confocal Microscope (Zeiss). Subsequently, the cocultures were processed for RNA isolation for transcript quantification of stem cells.

#### Immunostaining

The immunostaining of paraffin-embedded native tissue and organoids as well as whole mounts of cocultures was carried out as per established protocols.^4^^,^^25^ The antibodies used in this study are listed in Tables 1 and 2. Pericryptal cells were defined as mesenchymal cells immediately adjacent to the crypt base. Only fully longitudinal, clearly separated crypts were analyzed. The 10 cells surrounding the crypt base were counted for each crypt, with 10–20 crypts per mouse and multiple mice per group to account for biological variability. Marker-positive cells were recorded manually and expressed as a percentage of total pericryptal cells. The analyses were performed blinded to genotype or treatment.

**Table 1:** Primary Antibodies for Immunofluorescence Staining

Antibodies for immunofluorescence staining				
Antibody (clone)	Affinity	Dilution	Supplier	Catalog
Human/mouse active caspase-3	Rabbit, polyclonal	1:100	R&D Systems	AF835
E-Cadherin, clone 34	Mouse, monoclonal	1:100	BD Transduction Laboratories	610404
Alpha-smooth muscle actin, clone 1A4	Mouse, monoclonal	1:100	R&D Systems	MAB1420
Anti-vimentin antibody	Rabbit, polyclonal	1:100	Abcam	ab137321
Anti-FOXL1 antibody	Rabbit, polyclonal	1:50	Abcam	ab190226
Anti-GLi1 antibody	Rabbit, polyclonal	1:50	Rockland Antibodies and Assays	100-401-223
CD140a (PDGFRA) antibody, clone APA5	Mouse, Monoclonal	1:50	Thermo Fisher Scientific	14-1401-82
Integrin alpha 6 (ITGA6) antibody clone 6B4	Mouse, monoclonal	1:50	Acris Antibodies	BM6027P
Rspo3 antibody	Rabbit, polyclonal	1:50	Proteintech	17193-1-AP

**Table 2:** Primary Antibodies for Immunohistochemistry Staining

Antibodies for immunohistochemistry staining				
Antibody (clone)	Affinity	Dilution	Supplier	Catalog
Anti-MMP9 antibody	Rabbit, polyclonal	1:100	Sigma-Aldrich	AV33090
CD137 (4-1BB) antibody	Rabbit, polyclonal	1:100	Thermo Fisher Scientific	PA5-116949
CD200 antibody	Rabbit, polyclonal	1:100	Bioss Antibodies	BS-6030R
TRAF1 antibody	Rabbit, polyclonal	1:100	Thermo Fisher Scientific	BS-1212R

#### RNAscope in situ hybridization

In situ hybridization was performed according to ACD’s manual RNAscope protocol using the RNAscope 2.5 HD Detection Reagents-BROWN (ACD, cat# 322310). The single-plex RNAscope probes include mouse *Lgr5* (cat# 312171); mouse *Ephb3* (cat# 510251); mouse *Pdgfra* (cat# 480661); mouse *Gli1* (cat# 311001); mouse *Foxl1* (cat# 407401); and mouse *Rspo3* (cat# 402011). The mouse assay positive control included the RNAscope Control Slide-Mouse 3T3 Cell Pellet (cat# 310023).

#### Immunoblotting

Whole-cell lysates from native colonic tissue or primary mesenchymal cells of control or TNFR1^−/−^ animals were prepared as per established methods. The primary and secondary antibodies are listed in Table 3. Antibodies were detected using the LI-COR ODYSSEY 9120 infrared imaging system. Densitometric analyses of the band intensities were performed using ImageJ software^34^ (version 1.46; National Institutes of Health). The primary control colonic myofibroblasts were seeded at 1 × 10^3^ cells/mL in 6-well plates grown to 70% confluency and were treated with 10 ng/mL epidermal growth factor for 5 minutes and 20 minutes independently as positive controls for the activation of the PI3K and MAPK pathways. To determine the role of ITGA6 in regulating the proliferation and/or migration of TNFR1^−/−^ colonic myofibroblasts, a blocking antibody directed against ITGA6 was applied to TNFR1^−/−^ colonic myofibroblasts grown to 70% confluence (5 days). Because the cell-cycle inhibitor p27^Kip1^ regulates both fibroblast proliferation and migration,^35^^,^^36^ its expression was used as a functional readout. Dose escalation studies determined that 0.2 μg of anti-ITGA6 restored p27^Kip1^ expression to control levels (not shown). In other experiments, control or TNFR1^−/−^ colonic myofibroblasts were seeded at 1 × 10^3^ cells/mL in 24-well plates and at 70% confluency were treated with 0.2 μg/mL of anti-ITGA6 and IgG isotype control for 5 days for whole cell lysate preparation.

**Table 3:** Primary and Secondary Antibodies for Immunoblotting

Antibodies for immunoblotting and recombinant proteins				
Antibody (clone)	Affinity	Dilution	Supplier	Catalog
Anti-vimentin antibody	Rabbit, polyclonal	1:1000	Abcam	ab137321
Alpha-smooth muscle actin, clone 1A4	Mouse, monoclonal	1:1000	R&D Systems	MAB1420
Anti-FOXL1 antibody	Rabbit, polyclonal	1:500	Abcam	ab190226
Anti-GLi1 antibody	Rabbit, polyclonal	1:500	Rockland Antibodies and Assays	100-401-223
CD140a (PDGFRA) antibody, clone APA5	Mouse, monoclonal	1:500	Thermo Fisher Scientific	14-1401-82
p27/Kip1 antibody, clone 225,501	Mouse, monoclonal	1:1000	R&D Systems	MAB2256
Phospho-Akt (Ser473) antibody	Rabbit, polyclonal	1:1000	Cell Signaling	9271
Akt antibody, clone 40D4	Mouse, monoclonal	1:1000	Cell Signaling	2920
Phospho-p44/42 MAPK (Erk1/2) (Thr202/Tyr204), clone D13.14.4E XP antibody	Rabbit, monoclonal	1:1000	Cell Signaling	4370
p44/42 MAPK (Erk1/2) antibody	Rabbit, polyclonal	1:1000	Cell Signaling	9102
Recombinant Anti-RAP1GAP antibody, clone Y134	Rabbit, monoclonal	1:1000	Abcam	ab32373
Anti-RAP1 antibody, clone 4c8/1	Mouse, monoclonal	1:1000	Abcam	ab14404
Rspo3 antibody	Rabbit, polyclonal	1:500	Proteintech	17193-1-AP
Anti-β-actin antibody, clone AC-15	Mouse, monoclonal	1:5000	Millipore Sigma	A5441
GAPDH antibody	Rabbit, polyclonal	1:5000	Genetex	GTX100118
TNFRI/TNFRSF1A antibody	Mouse, monoclonal	1:1000	R&D Systems	MAB225
Phospho-IκBα (Ser32) (14D4) antibody	Rabbit, monoclonal	1:1000	Cell Signaling	2859
IκBα antibody	Rabbit, monoclonal	1:1000	Cell Signaling	9242
IRDye 800CW IgG secondary antibody	Donkey anti-rabbit	1:10,000	LICOR	926-32213
IRDye 680RD IgG secondary antibody	Goat anti-mouse	1:10,000	LICOR	926-68070
Anti-integrin α6 antibody, clone NKI-GoH3	Rabbit, monoclonal	0.2 μg	Millipore Sigma	MAB1378
IgG control	Rabbit, polyclonal	0.2 μg	Millipore Sigma	12-370
TNFR1/CD120a/TNFRSF1A neutralizing antibody	Rabbit, monoclonal	0.5 μg	SinoBiological	150496-M08H
IgG control	Rabbit, polyclonal	0.5 μg	SinoBiological	CR1
R-Spondin 3 neutralizing antibody	Sheep, polyclonal	0.05 μg	R&D Systems	AF35001
IgG control	Sheep, polyclonal	0.05 μg	R&D Systems	5-001-A
TNFR2 recombinant antibody (ARC0397)	Rabbit, monoclonal	1:1000	Thermo Fisher Scientific	MA5-35276
Recombinant R-Spondin 3 protein	CHO-derived mouse protein	5 ng	R&D Systems	4120-RS

#### Wound healing assay

Wounds ∼1 mm in width were created by scratching confluent monolayers of control or TNFR1^−/−^ primary colonic myofibroblasts with pipette tips obliquely cut (45° angle) with a sterile razor blade. Cells in suspension were removed by aspiration with PBS. Phase contrast images were taken at 0, 2, 4, 16, and 24 hours, after scratching, using a Leica DM IL LED inverted microscope (Leica) equipped with a 5×/0.12 NA objective and a digital camera. The wound closure areas were measured with ImageJ by subtracting the total amount (A) of grayscale pixel counted in the cell-free area remaining after 24 hours from the initial wound area; hence: “Relative wound area” [pixel] = A_initial-_A_T_ hrs. For antibody blocking experiments, control or TNFR1^−/−^ colonic myofibroblasts were seeded at a density of 1 × 10^3^ cells/mL in 24-well plates and treated with an anti-ITGA6 antibody and its IgG isotype control at a concentration of 0.2 μg/mL for 0 to 16 hours at passage.

#### RNA isolation, complementary DNA synthesis, and quantitative real-time polymerase chain reaction

Total RNA from the native tissue or primary colonic myofibroblasts was extracted using the Ambion PureLink RNA Isolation Kit (Thermo Fisher Scientific, cat# 12183025) according to the manufacturer’s protocol. Complementary DNA (cDNA) was synthesized from 50 to 300 μg of treated RNA for each sample using Verso cDNA Synthesis Kit (Thermo Fisher Scientific, cat# AB1453B) according to the manufacturer’s recommendations using a CFX384 Touch Real-Time PCR Detection System (Bio-Rad). Quantitative polymerase chain reaction (PCR) for a subset of primers was performed using the TaqMan Fast Advanced Master Mix (Thermo Fisher Scientific, cat# 4444557) on a Bio-Rad iQ5 thermocycler. The primer and probe oligonucleotides were obtained from Integrated DNA Technologies (*Lgr5*: [Mm.PT.58.12492947]; *Ephb3*: [Mm.PT.58.30165903], and *Actin*: [Mm.PT.39a.22214843]). All other quantitative PCR reactions were performed in duplicate using 5.2 μL SYBR Premix Ex Taq II (Tli RNase H Plus), ROX Plus (Takara, cat. no. RR82WR), 0.2 μL each of sense and antisense primers (Table 4) at 10 μM, and 2 μL cDNA. No-reaction controls contained 2.4 μL nuclease-free water instead of cDNA, giving a final reaction volume of 10 μL. The samples were heated to 95 °C for 3 minutes followed by 37 cycles of 94 °C for 15 seconds, 54 °C for 20 seconds, and 72 °C for 25 seconds. The primers used in this study are listed in Table 4. The quantitative real-time PCR data were analyzed by using the comparative ΔΔCt method.^37^

**Table 4:** Forward and Reverse Primer Sequences for SYBR Quantitative Polymerase Chain Reactions

SYBR forward and reverse primers		
Gene	Forward	Reverse
m-Wnt5A	CAACTGGCAGGACTTTCTCAA	CATCTCCGATGCCGGAACT
m-Grem1	AGACCTGGAGACCCAGAGTA	GTGTATGCGGTGCGATTCAT
m-Wnt2b	GGGGATGTTGTCACAGATCA	CTGCTGCTGCTACTCCTGACT
m-Bmp4	TGGGCTGGAATGATTGGATT	CAGTCCCCATGGCAGTAGAAG
m-Bmp2	GGGACCCGCTGTCTTCTAGT	TCAACTCAAATTCGCTGAGGAC
m-Rspo3	TTGACAGTTGCCCAGAAGGG	CTGGCCTCACAGTGTACAATACT
m-Fgf-2	GGCTGCTGGCTTCTAAGTGTG	TTCCGTGACCGGTAAGTATTG
m-αSMA	GCATCCACGAAACCACCTA	CACGAGTAACAAATCAAAGC
m-Gli1	AGCCTGAGTCTGTGTATG	CACCACCAGCATGTATTG
m-Foxl1	TGCCGCATTCCACAGCATAGTC	CAAAGTGAGTTCCAGGACAGCCAG
m-Vim	CGGCTGCGAGAGAAATTGC	CCACTTTCCGTTCAAGGTCAAG
m-Pdgfrα	GCAGTTGCCTTACGACTCCAGA	GGTTTGAGCATCTTCACAGCCAC
m-Mmp9	GCTGACTACGATAAGGACGGCA	TAGTGGTGCAGGCAGAGTAGGA
h-β-ACTIN	AGCACGGCATCGTCACCAACT	TGGCTGGGGTGTTGAAGGTCT
h-ITGA6	GAGCTTTTGTGATGGGCGATT	CTCTCCACCAACTTCATAAGGC
h-PDGFRα	AACCGTGTATAAGTCAGGGGA	ATTTCTTCCAGCATTGTGAT
h-RSPO3	CAGAATCGCATGACCCAC	AGCAGCCAGAAGCCAGAG

#### Human primary myofibroblast culture and anti–tumor necrosis factor receptor 1 experiments

The normal human primary colonic myofibroblast cell line, CCD-18Co (ATCC, cat# CRL-1459) was cultured in Eagle’s Minimum Essential Medium (ATCC, 30-2003), supplemented with nonessential amino acids, 2 mM L-glutamine, 1 mM sodium pyruvate, and 1500 mg/L sodium bicarbonate and 10% FBS with 1% penicillin–streptomycin (10,000 U/mL) and maintained at 37 °C in a 5% CO_2_/air mix. The cultures were passaged every 5 days at a ratio of 1:3. To determine the optimal concentration for blocking TNFR1-mediated signaling in human mesenchymal cells, preliminary experiments using human control organoids determined that, in response to TNF, epithelial Iκβα is degraded at the highest concentration of 100 ng TNF at 1 hour (not shown). H-organoids subsequently exposed to increasing doses of H-anti–TNFR1 demonstrated that the levels of Iκβα degradation were restricted in a dose-dependent manner, with little to no effect on the total levels of TNFR1, relative to the IgG isotype control (not shown). Therefore, anti-TNFR1 at a concentration of 0.5 μg/mL effectively blocks TNFR1-mediated signaling. Thus, 18Co cells were treated with an anti-TNFR1 antibody and its IgG isotype control at a concentration of 0.5 μg/mL for 5 days at passage. The cells were seeded at a density of 1 × 10^3^ cells/mL in 24-well plates in parallel for immunostaining and RNA collection.

#### 5-Ethynyl-2′-deoxyuridine assay for proliferation

Whole mounts of organoids or cocultures were stained with the Click-iT Plus EdU Alexa Fluor488 Imaging Kit (ThermoFisher Scientific, cat# C10632) as per the manufacturer’s recommendations. For antibody blocking experiments, control or TNFR1^−/−^ colonic myofibroblasts were seeded at a density of 1 × 10^3^ cells/mL in 24-well plates and treated with an anti-ITGA6 antibody and its IgG isotype control at a concentration of 0.2 μg/mL for 72 hours at passage.

#### RNA sequencing

The control (TNFR1^+/−^) and TNFR1^−/−^ primary colonic myofibroblasts (passage 6–8) were seeded at a density of 1 × 10^3^ cells/50 μL of Matrigel into 24-well plates and were grown to 70% confluency. At day 5, the colonic myofibroblasts were extracted from Matrigel after a brief incubation at 4 °C via centrifugation at 1200 rpm for 5 minutes. The total RNA from primary colonic myofibroblasts was extracted using the Ambion PureLink RNA Isolation Kit (Thermo Fisher Scientific, cat# 12183025) according to the manufacturer’s protocol. mRNA transcripts from control and TNFR1^−/−^ colonic myofibroblasts were prepared for 2 × 75 bp sequencing on a HiSeq 4000 (Illumina). The library preparation and sequencing were performed by Children's Hospital Los Angeles Single Cell, Sequencing, and CyTOF Core Laboratory. The reads were first mapped to the latest UCSC transcript set using Bowtie2 version 2.1.0, and the gene expression level was estimated using RSEM v1.2.15. The trimmed mean of M-values method was used for the normalization of the raw count. Differentially expressed genes were identified using the edgeR program. Genes showing altered expression with *P* < .05 and more than 1.5-fold changes were considered differentially expressed. Cluster profiler was used for the Gene Ontology and pathway enrichment analysis.

#### Single-cell RNA sequencing

Control (TNFR1^+/−^) and TNFR1^−/−^ primary colonic myofibroblasts were prepared in a similar manner to RNA-seq. Following centrifugation, cells were reconstituted in 10 mL of HEPES-buffered saline and 1% bovine serum albumin solution and were passed through a 40-μm filter to suspend into single cells. To remove dead cells for the final flow, single-cell suspensions of control or TNFR1^−/−^ colonic myofibroblasts were stained with the EasySep Dead Cell Removal (Annexin V) Kit (STEMCELL, cat# 17899) according to the manufacturer's guidelines. The live cells were quantified and reconstituted in 100 to 200 μL of HEPES-buffered saline and 1% bovine serum albumin solution. The samples with live cell counts of 95% and above only were processed for scRNA-seq. The transcript abundance measurements were normalized by the per-cell read total, log-transformed, and collapsed to principal components. The top 50 principal components were used for UMAP. The UMAP was used for clustering. Clustering was performed with the Leiden algorithm with a resolution parameter of 1e-5. The markers of each cluster were identified using the Jensen-Shannon specificity score. The significance of each marker was computed via linear regression, which provided an r^2^ value that could be used to screen the initial gene lists.

For pathway enrichment analysis, the transcript abundances of each cluster were collapsed as a per-cluster, per-gene mean. Genes with an expression level <0.2 were filtered out. Markers of each cluster were defined as a set of genes whose cluster expression was >2 times higher than their next highest expression level among the cell clusters. The resulting matrix file was used as input into enrichr. Because clusters 2 and 4 did not exhibit markers of enriched gene expression, these clusters were excluded from enrichr. The search for enriched pathways was run against the Kyoto Encyclopedia of Genes and Genomes and MSigDB HALLMARK target databases. Pathways with an enrichment-adjusted *P* value (q value) < .01 were considered significant and displayed.

### Statistical Analysis

GraphPad Prism software was used for statistical analyses. Data are presented as means with standard error of the mean. Analysis was carried out by either an unpaired Student *t* test or a 1-way analysis of variance, with a Šídáks post-hoc correction, as indicated in figure legends, where ∗*P* < .05; ∗∗*P* < .01; ∗∗∗*P* < .001; ∗∗∗∗ < .0001; and ^#^*P* = .05 to .09.

## CRediT Authorship Contributions

Safina Gadeock, PhD (Conceptualization: Lead; Data curation: Lead; Formal analysis: Lead; Funding acquisition: Supporting; Investigation: Lead; Methodology: Lead; Resources: Supporting; Validation: Lead; Visualization: Lead; Writing – original draft: Lead; Writing – review & editing: Lead)

Nandini Girish (Data curation: Equal; Investigation: Supporting; Methodology: Equal; Project administration: Lead; Validation: Equal; Visualization: Supporting)

Cambrian Y. Liu (Conceptualization: Equal; Formal analysis: Supporting; Funding acquisition: Equal; Methodology: Supporting; Resources: Supporting; Software: Lead; Supervision: Equal; Validation: Supporting; Writing – review & editing: Equal)

Ying Huang (Data curation: Supporting; Methodology: Supporting; Resources: Supporting)

Barun Das (Methodology: Supporting)

Tracy C. Grikscheit (Data curation: Supporting)

D. Brent Polk (Conceptualization: Lead; Funding acquisition: Lead; Project administration: Lead; Resources: Lead; Supervision: Lead; Writing – review & editing: Lead)
